# Non-autoimmune Familial Hyperthyroidism in Children: The Importance of Genetic Testing

**DOI:** 10.7759/cureus.100624

**Published:** 2026-01-02

**Authors:** Mariana Sa Pinto, Tomás Ferrão, Andreia Dias Preda, Margarida Moreno Fernandes, Maria Adriana Rangel

**Affiliations:** 1 Pediatrics and Neonatology, Unidade Local de Saúde Gaia - Espinho, Vila Nova de Gaia, PRT; 2 Pediatrics, Unidade Local de Saúde da Região de Aveiro, Aveiro, PRT; 3 Pediatric Endocrinology, Unidade Local de Saúde Gaia - Espinho, Vila Nova de Gaia, PRT

**Keywords:** adolescent, genetic testing, hereditary diseases, hyperthyroidism, thyrotropin receptor

## Abstract

Hyperthyroidism of non-autoimmune etiology is an uncommon occurrence, and its etiology may be the result of germline variants capable of activating the thyroid-stimulating hormone receptor (TSHR). The case of a 16-year-old adolescent with a family history of hyperthyroidism and negative antithyroid antibodies is presented. Imaging revealed bilateral subcentimeter nodules, and thyroid scintigraphy showed normal, heterogeneous uptake, with no hyperfunctioning areas. Genetic testing identified the likely pathogenic variant c.2009A>G p.(Asn670Ser) in the TSHR gene, which has been previously described in families with non-autoimmune hyperthyroidism. The patient is undergoing treatment with methimazole, with normalization of thyroid function and clinical surveillance.

This case highlights the need to consider alternative etiologies of hyperthyroidism, particularly in the absence of autoimmune markers and in the presence of a strong family history, reinforcing the role of genetic testing in this clinical context.

## Introduction

The predominant etiology of hyperthyroidism in children is autoimmune disease, particularly Graves' disease, accounting for approximately 80%-95% of cases [1-3]; however, in rare instances, hyperthyroidism may have a genetic basis due to activating germline variants of the thyroid-stimulating hormone receptor (TSHR) gene. This form of hyperthyroidism lacks autoimmune features and is transmitted in an autosomal dominant pattern, resulting in familial clustering [1,2]. It is characterized by persistent thyrotoxicosis, negative TSHR antibodies (TRAbs), a positive family history, and may be associated with nodular hyperplasia [1,3-5]. Recognizing these hereditary forms is essential for accurate diagnosis in pediatric patients, as well as for individualized therapeutic planning and appropriate genetic counseling [1,3-5].

## Case presentation

A 16-year-old male adolescent, without siblings, was referred to the pediatric endocrinology outpatient clinic due to palpitations and suspected hyperthyroidism. Family history was significant for hyperthyroidism in the father, paternal grandfather, and paternal uncle, all of whom had undergone thyroidectomy. The father’s disease course was difficult to control with antithyroid drugs, requiring frequent dose adjustments and intermittent use of a block-and-replace strategy.

The patient denied weight loss, gastrointestinal disturbances, insomnia, decline in school performance, or ocular symptoms, such as exophthalmos. Laboratory evaluation demonstrated persistently suppressed TSH levels, with elevated free thyroxine (free T4) and negative TRAbs. A detailed summary of laboratory findings is presented in Table 1.

**Table 1:** Thyroid function tests at diagnosis TSH: thyroid-stimulating hormone; free T4: free thyroxine; TRAbs: thyroid-stimulating hormone receptor antibodies

Laboratory parameter	Patient value	Reference range	Units
TSH	<0.005	0.4-4.0	μIU/mL
Free T4	2.1	0.8-1.8	ng/dL
TRAbs	Negative	Negative	-

**Figure 1:**
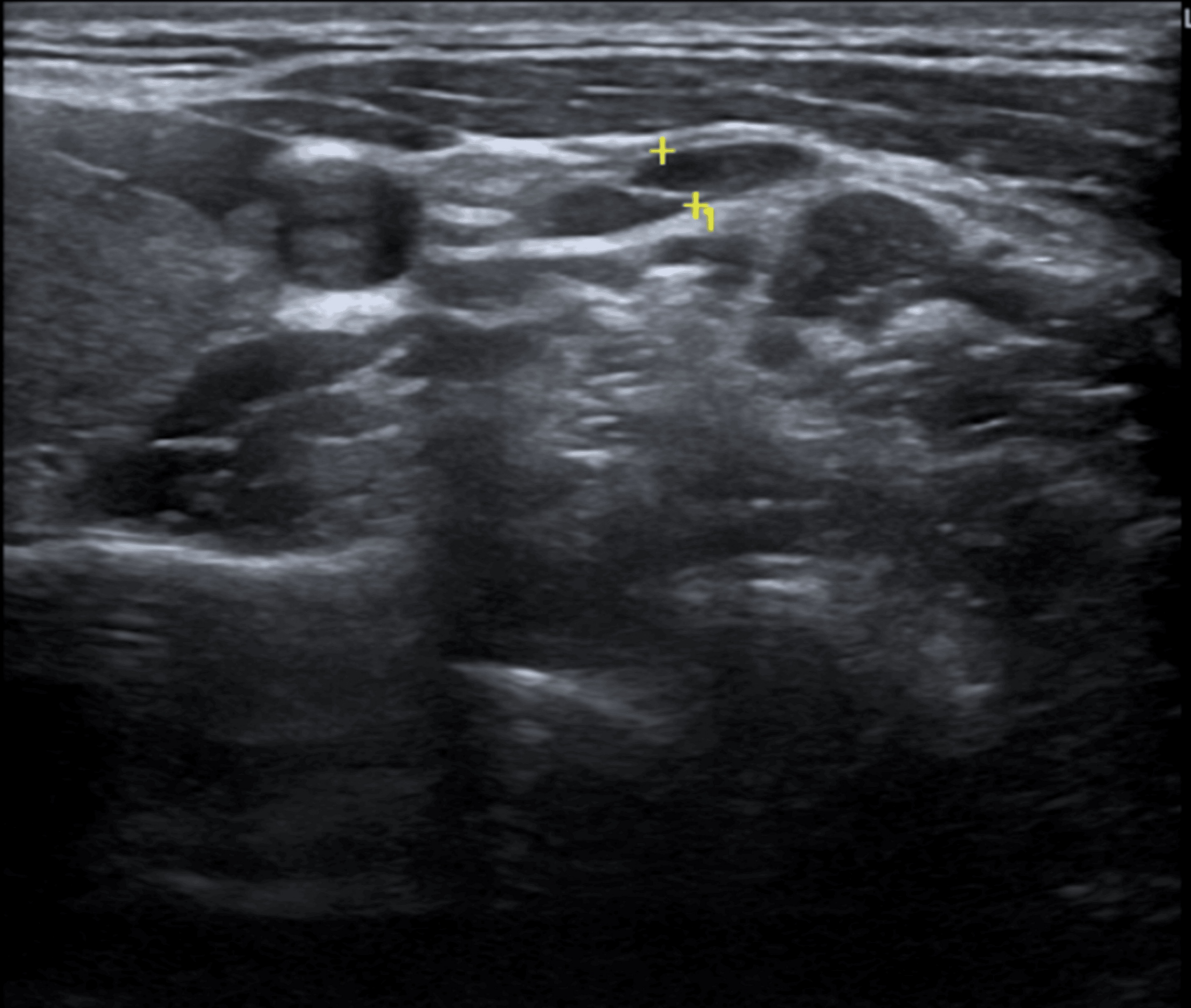
Thyroid ultrasound imaging

Thyroid ultrasound revealed a mildly enlarged gland with bilateral nodules measuring up to 8 mm, classified as TI-RADS 3 and 4, without suspicious malignant features or significant interval growth (Figure 1). Thyroid scintigraphy demonstrated normal global uptake (2.4%), with no hyperfunctioning nodules.

Treatment with methimazole (0.15 mg/kg/day) was initiated, leading to progressive normalization of thyroid function. The patient is currently maintained on a low dose (2.5 mg/day), remains asymptomatic, and reports no adverse effects. Genetic testing identified a likely pathogenic heterozygous TSHR variant, c.2009A>G p.(Asn670Ser), previously associated with familial non-autoimmune hyperthyroidism and nodular hyperplasia. The father is awaiting genetic testing.

## Discussion

This case illustrates persistent hyperthyroidism with negative thyroid autoantibodies and a positive family history, a clinical profile that is highly suggestive of familial non-autoimmune hyperthyroidism [1,2,5]. The p.Asn670Ser variant in the TSHR gene is a constitutively activating variant, previously associated with early-onset thyrotoxicosis and, in some cases, thyroid nodules [1,2,4]. Distinguishing this condition from Graves' disease is essential, as prognosis, risk of recurrence, and long-term management differ substantially [2,3,6]. Antithyroid drugs may achieve initial biochemical control; however, in genetically mediated hyperthyroidism, they are generally considered a temporizing measure rather than a curative option, with definitive therapies, such as radioactive iodine or thyroidectomy, often required [2,3,5].

This case highlights the importance of early recognition and thorough evaluation of family history, as well as the role of genetic testing in enabling timely diagnosis and preventive genetic counseling in affected families, particularly given the autosomal dominant pattern of transmission and the associated risk to relatives [1,3,5].

## Conclusions

Familial non-autoimmune hyperthyroidism represents a rare but clinically significant cause of persistent thyrotoxicosis in pediatric patients, particularly in the presence of negative thyroid autoantibodies and a suggestive family history, where early identification of an activating germline TSHR variant enables accurate diagnosis, individualized therapeutic planning, and appropriate genetic counseling. Early genetic diagnosis also influences long-term management decisions, including the timing of definitive therapy. Identification of a pathogenic TSHR variant supports a family-centered approach, including cascade genetic screening of at-risk relatives, as a preventive strategy to optimize long-term follow-up and management.
